# The impact of meteorological parameters on the number of applications to the emergency department with acute urticaria: A retrospective study

**DOI:** 10.1371/journal.pone.0290535

**Published:** 2023-09-13

**Authors:** Orhan Delice, Sibel Güçlü Utlu, Şenol Arslan, Halil Ibrahim Doru, Murat Daş

**Affiliations:** 1 Department of Emergency Medicine, Erzurum Training and Research Hospital, Erzurum, Turkey; 2 Department of Emergency Medicine, Çanakkale Onsekiz Mart University, Çanakkale, Turkey; University 20 Aout 1955 skikda, Algeria, ALGERIA

## Abstract

The study aimed to investigate the relationship between the patients who applied to the emergency department with acute urticarial and meteorological factors and determine the associated weather conditions. The relationship between acute urticaria patients who applied to the emergency department of a training and research hospital in a city with high altitude and continental climate characteristics in the eastern part of Turkey between January 2019 and December 2019 and meteorological data was evaluated retrospectively. The necessary data for the weather were obtained from the data of the Erzurum Meteorology Directorate, and the patient data were obtained from the hospital information management system and patient files. The meteorological data of the patients at the date of admission and the characteristics of the patients at that date were compared. The study identified 691 patients who applied to the emergency department diagnosed with urticaria in 2019. According to the seasons, it was observed that the patients applied most frequently in the summer months [n = 239; 34.6%]. In the univariable regression model, which was created by taking the values of weather events as a reference on the days when the urticaria presentation was not observed (Group I), it was determined that every 1-hour increase in the sunshine hour increased the probability of urticaria by 7.4% (p = 0.018). When the meteorological parameters on the days without urticaria (Group I) and the days with urticaria presentation (Group II) were compared, the sunshine hours were 7.9 (4.0–10.6) hours in Group II and 6.65 (3.3–8.85) hours in Group I (p = 0.001). Regarding relative humidity, higher humidity rates were observed in Group I compared to Group II (p = 0.009). In terms of mean temperature, daily maximum, and minimum temperature, higher temperature rates were detected in Group II (p<0.001). A relationship was determined between urticaria attacks and relative humidity and daily maximum and minimum temperature in patients who applied to the emergency department with acute urticaria.

## 1. Introduction

Urticaria is defined as the sudden development of transient hives (wheals) and angioedema or both. A wheal is characterized by a circumscribed superficial edema of the skin, mostly surrounded by a bright red erythema and associated with a strong itching or burning sensation [1].

Patients often apply to emergency departments because of the sudden onset of uncomfortable skin lesions and the possibility of developing life-threatening anaphylaxis. Acute urticaria is the most common skin condition leading to emergency room visits in both adults and children [2, 3]. Acute urticaria is common, and approximately 10–20% of the population is affected at some point in their lives [4]. One-third of patients present with both urticaria and angioedema, 30% to 40% with isolated urticaria, and 10% to 20% with isolated angioedema [5]. Because urticaria can impair quality of life, it also has social importance because it can affect work or school performance.

Acute urticaria is defined as the occurrence of papules, angioedema, or both for 6 weeks or less. More than 50% of acute urticaria cases have triggering factors. The most common triggers are infections (40%), drugs (9.2%), and food (0.9%). The most common drugs held responsible are angiotensin-converting enzyme (ACE) inhibitors and nonsteroidal anti-inflammatory drugs (NSAIDs); however, approximately 50% of the triggering factor remains idiopathic [6]. ACE inhibitors are more often responsible for bradikininergic angioedema [6]. Physical urticaria 0.5% of the population is thought to suffer from chronic physical urticaria, and these conditions constitute 15–25% of chronic urticaria [7]. Certain subtypes of physical urticaria caused by mechanical, thermal (cold or hot), or electromagnetic waves [8].

Urticaria is caused by either immunoglobulin E-mediated or non-immunoglobulin E-mediated release of other inflammatory mediators—mainly histamine—from mast cells and basophils [9].

We saw that on some days there was no admission to the emergency department because of acute urticaria, and on some days many patients were admitted. We thought that the weather events that society is exposed to on days with many patient admissions may be effective. In fact, about half of the factors triggering acute urticaria could not be detected. Many studies have shown that some diseases are associated with weather events. For example, low average temperatures, low sunlight, high humidity, and high wind speeds have been found to increase the risk of stroke [10].

With the hypothesis that weather events may cause an increase in allergic reactions, this study aimed primarily to evaluate the effect of weather and climate parameters on the formation of urticaria and to investigate whether meteorological parameters affect the number of patients presenting with urticaria.

## 2. Materials and methods

### 2.1. Patient population and methods

Patients over the age of 18 who were diagnosed with acute urticaria between January 1, 2019, and December 31, 2019, in the emergency department of a hospital in eastern Turkey, were included in the study. Patients with chronic diseases and a history of drug use related to these diseases were excluded. Prior to the study, local ethics committee approval was obtained (dated 03.01.2022 and numbered 2021/23–296). Patient data were obtained with the hospital information management system and archive files. Demographic data such as age, gender, admission dates, and meteorological factors at that time were used.

**Fig 1:**
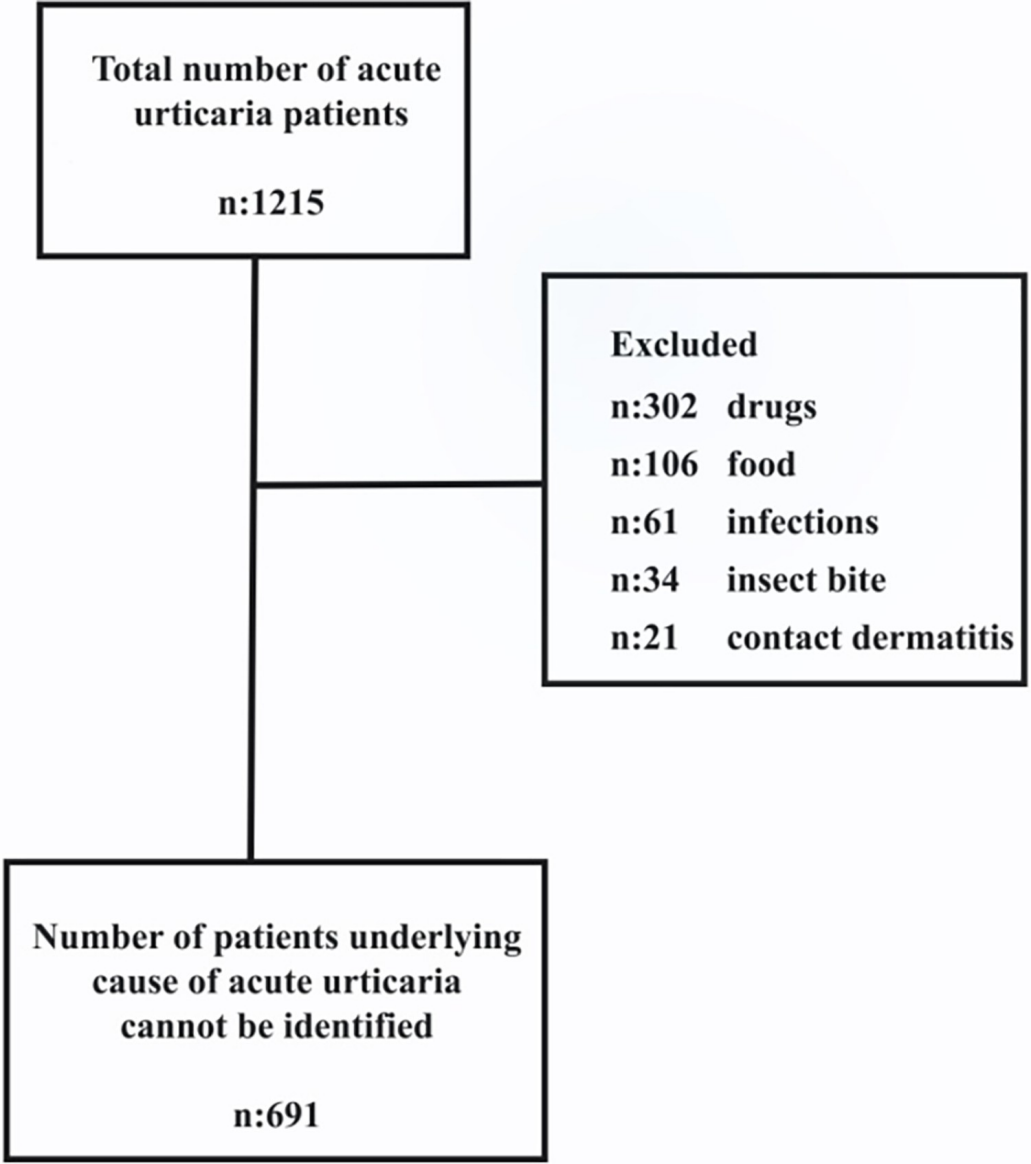
Number of patients excluded and included in acute urticaria patients.

Diagnoses used for screening acute urticaria according to ICD-10 (International Statistical Classification of Diseases and Related Health Problems) of L50.0 (Allergic Urticaria), L50.1 (Idiopathic Urticaria), L50.2 (Cold and Hot Urticaria), L50.3 (Dermatographic Urticaria), L50.4 (Vibratory Urticaria), L50.5 (Cholinergic Urticaria), L50.6 (Contact Urticaria), L50.8 (Chronic or Recurrent Urticaria), and L50.9 (Unspecified Urticaria) were used. Meteorological data on days without urticaria applications were defined as Group I, and meteorological data on days with urticaria applications were defined as Group II. The data available in Group II were divided into two further subgroups, according to the number of patients admitted: Group IIA for days with 1–4 patient admissions and Group IIB for days with 5 or more applications. When the total number of patients was evaluated, 1215 patients were diagnosed with acute urticaria. Starting January 1, the data of patients diagnosed with acute urticaria were obtained by scanning the hospital information management system. Patients who presented to the emergency department with acute urticaria and whose trigger factors were known or who were diagnosed with physical urticaria were excluded from the study. We did not have a patient who was diagnosed with urticaria because of physical factors. The excluded factors are shown in Fig 1.

### 2.2. Meteorological parameters

The parameters were obtained from the data of the 12th Regional Directorate of Meteorology and used. Among the meteorological parameters, sunshine duration (hours), sunshine intensity (kilowatt/square meter = Kwatt/m^2^), pressure (hectopascal = hPa), cloudiness (8 Octa), wind speed (meter/second = m/s), relative humidity (%), and temperature (degrees Celsius = °C) were evaluated for use in the study. These parameters were used as the daily average, maximum, and minimum values.

### 2.3. Statistical analysis

In descriptive statistics, numerical data were expressed as a median and interquartile range and categorical data as numbers and percentages. The normality test of numerical data was analyzed with the Shapiro–Wilk test. The significance of the difference between the groups in terms of the median values was analyzed using the Mann-Whitney U test. The comparison of Group I, Group IIA, and Group IIB was performed with the Kruskall Wallis test. Odds ratios (ORs; 95% confidence intervals [CI]) of the independent meteorological variables were calculated with univariate and multiple logistic regression models to predict Group II. A multivariate logistic regression analysis was created by performing stepwise variable selection on those variables with a univariate p-value of <0.25. Multinomial logistic regression analysis was used to identify the effects of each meteorological parameters. All statistical analyses were conducted using SPSS 19.0 for Windows (IBM Corp., Armonk, New York, USA) and R software version 3.6.2. All p-values of less than 0.05 were considered to indicate statistical significance.

## 3. Results

The total number of patients admitted to our emergency department in 2019 was 239,271. Acute urticaria patients constituted approximately 0.5% of the applications.The seasonal (winter, spring, summer and autumn) percentages of total admissions to the emergency department were similar to each other (24.03%, 25.12%, 24.60% and 26.24%, respectively). Percentage of patients with acute urticaria of unknown origin were highest in summer (June, July, and August; n = 239; 34.6%) and lowest in winter (December, January and February; n = 111; 16.1%) [Table 1].

**Table 1:** Demographic parameters (n = 691).

Age	35.0 (25.0–46.0)
Gender	
Female	401 (58.0)
Male	290 (42.0)
Number of patients by seasons	
Winter	111 (16.1)
Spring	171 (24.7)
Summer	239 (34.6)
Fall	170 (24.6)
Sunshine (hours)	4.2 (0.7–8.7)
Daily Average Actual Pressure (hPa)	811.7 (809.0–814.1)
Daily Average Cloudiness (8 Octa)	5.1 (3.3–6.5)
Daily Average Relative Humidity (%)	74.2 (60.0–81.5)
Daily Average Wind Speed (m/s)	1.1 (0.7–1.6)
Daily Average Temperature (°C)	2.6 [(-1.6)-(14.0)]
Daily Total Global Insolation Radiation (Kwatt/m^2^)	258.6 (144.3–390.3)
Number of days with urticaria patient n (%)	276 (75.6)

**Fig 2:**
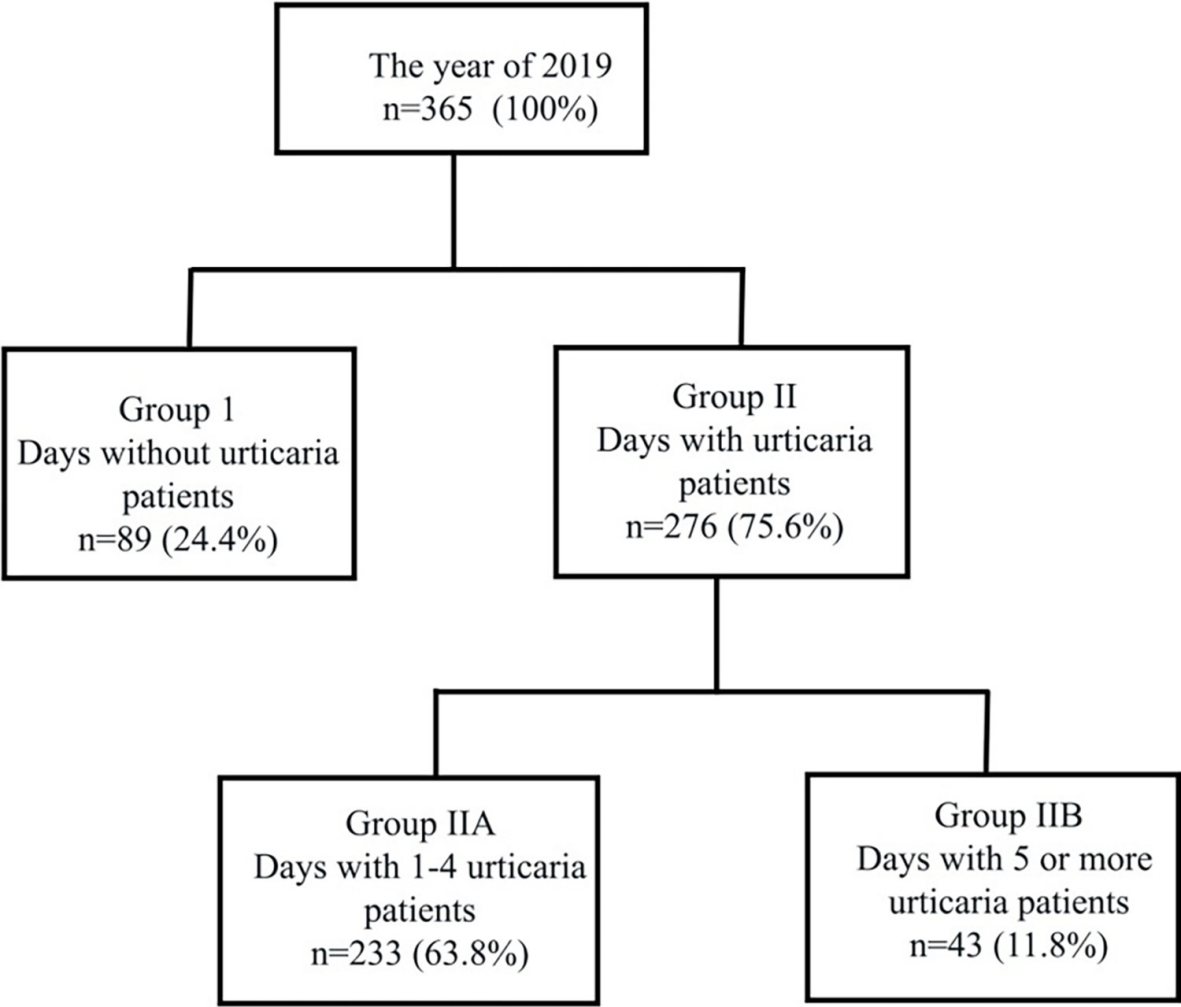
Grouping of urticaria patients admitted to the emergency department in 2019.

In our study, 691 (56.8%) patients in whom the triggering factor could not be determined were identified among 1215 patients who applied to the emergency department with the diagnosis of acute urticaria in 2019. Of these, 58% were female, 42% were male, and the median age of the patients was 34.5 (25.0–46.0) years. In 2019, the number of days with admission to the emergency department with the complaint of urticaria was 276 (75.6%). In Fig 2, the number of urticaria admission days is shown by grouping.

Table 1 summarizes the median and 25–75% quartiles of sunshine hours, daily average temperature, and other meteorological parameters for 2019.

When the meteorological parameters on Group I days were compared with those on Group II days, the sunshine hours were 7.9 (4.0–10.6) hours in Group II and 6.65 (3.3–8.85) hours in Group I (p = 0.001). In terms of relative humidity, higher humidity rates were observed in Group I compared to Group II (p = 0.009). Regarding mean temperature and daily maximum and minimum temperatures, higher temperature rates were detected in Group II (p<0.001). The comparisons of other meteorological data are presented in Table 2.

**Table 2:** Comparison of days according to urticaria application status.

	Group I	Group II	p-value
	n = 89 (24.4%)	n = 276 (75.6%)	
Sunshine (hours)	6.65 (3.3–8.85)	7.9 (4.0–10.6)	0.009
Daily Max Actual Pressure (hPa)	814.9 (812.1–817.3)	814.6 (812.2–817.1)	0.851
Daily Average Actual Pressure (hPa)	813.6 (810.4–816.3)	813.1 (810.6–815.7)	0.652
Daily Min Actual Pressure (hPa)	811.9 (808.6–814.8)	811.5 (809.0–814.4)	0.558
Daily Average Cloudiness (8 Octa)	2.9 (1.2–5.3)	2.5 (0.9–4.9)	0.164
Daily Average Relative Humidity (%)	65.9 (49.9–76.8)	57.3 (43.8–74.1)	0.009
Daily Average Wind Speed (m/s)	0.85 (0.5–1.5)	1.1 (0.6–1.6)	0.033
Daily Max Temperature (°C)	7.5 (1.1–20.6)	16.8 (4.4–25.9)	<0.001
Daily Average Temperature (°C)	2.0 [(-3.1)-10.7]	10.5[(-0.2)-17.8]	<0.001
Daily Min Temperature (°C)	-1.75 [(-7.4)-4.4]	3.7 [(-3.5)-10.2]	<0.001
Daily Total Global Insolation Radiation (Kwatt/m^2^)	195.6 (124.8–297.6)	278.7 (158.4–404.4)	0.001

In the univariable regression model, which was created by taking the values of the weather events on the days without urticaria (Group I) as a reference, it was determined that every 1-hour increase in the sunshine hours increased the probability of urticaria by 1.074 [(1.012–1.139) p = 0.018]. Furthermore, each 1-degree rise in temperature predicted Group II by 1.056 (1.029–1.083). The power of other meteorological variables to predict Group II was not statistically significant (Table 3). The multivariable regression model revealed that the temperature increase was effective in predicting Group II [1.071 (1.029–1.115), (p = 0.001)].

**Table 3:** Logistic regression analysis of weather events predicting the probability of urticaria.

	Univariable analysis	p-value	Multivariable analysis	p-value
	OR (95% CI)		OR (95% CI)	
Sunshine (hours)	1.074 (1.012–1.139)	0.018		
Daily Average Actual Pressure (hPa)	0.983 (0.929–1.041)	0.563		
Daily Average Cloudiness (8 Octa)	0.166 (0.838–1.031)	0.166		
Daily Average Relative Humidity (%)	0.983 (0.969–0.996)	0.012		
Daily Average Wind Speed (m/s)	1.417 (0.996–2.015)	0.053		
Daily Average Temperature (°C)	1.056 (1.029–1.083)	<0.001	1.071 (1.029–1.115)	0.001
Daily Total Global Insolation Radiation (Kwatt/m^2^)	1.974 (1.217–3.202)	0.006		

**Fig 3:**
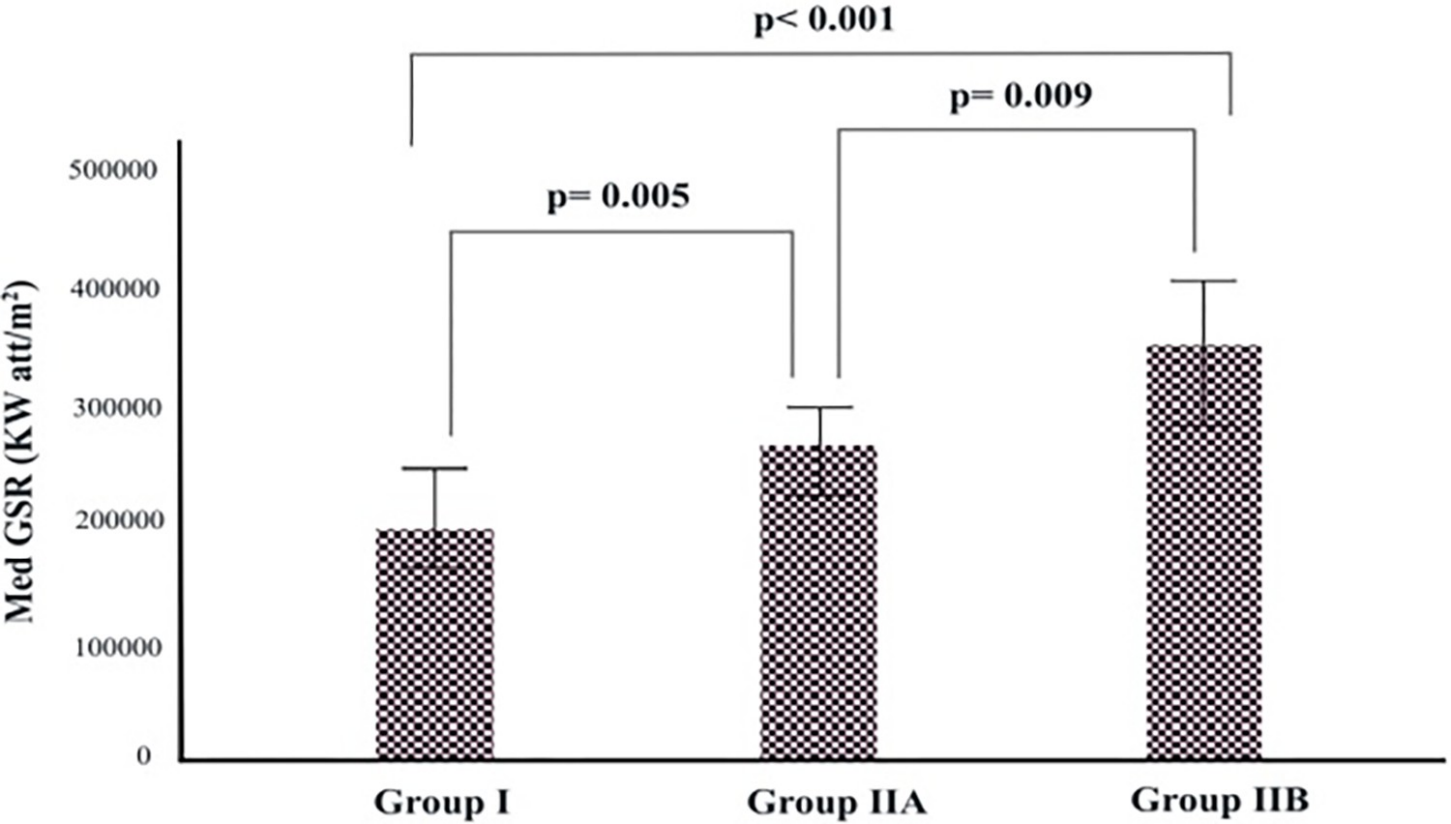
Distribution of median daily total global sunshine radiation by groups. GSR; Global solar radiation.

**Fig 4:**
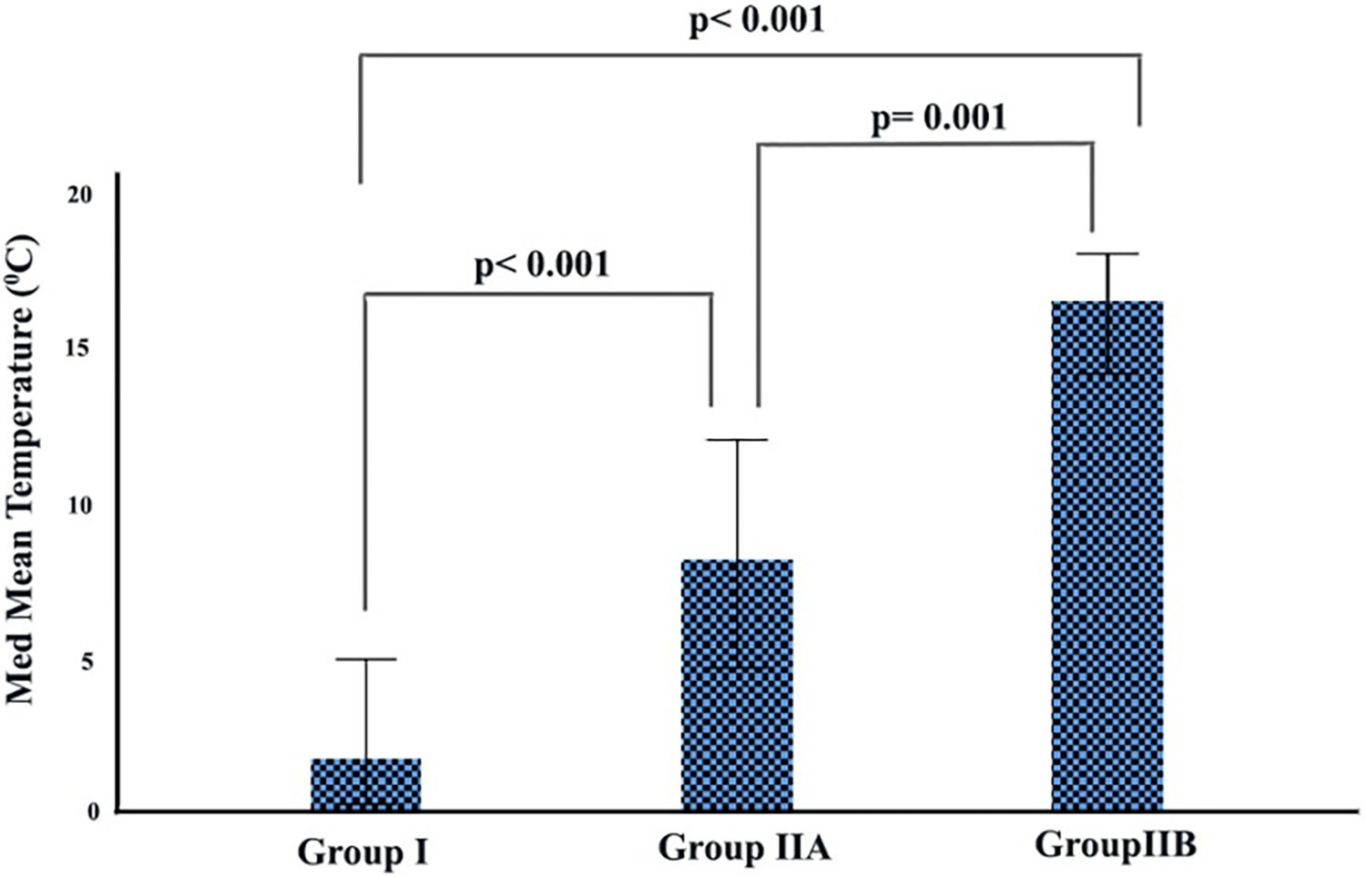
Distribution of daily average temperatures by groups.

The median daily total global solar radiation (GSR) and temperatures of Group I, Group IIA, and Group IIB are summarized in Figs 3 and 4. Accordingly, the median GSR and temperature increase gradually increased from Group I to Group IIB, and the difference between them was statistically significant (p<0.001 and p<0.001, respectively).

The multinominal logistic regression model created with reference to Group I, estimating Group IIA and Group IIB, was analyzed. In the univariable analysis, the odds ratio for each 1-hour increase in sun exposure to predict Group IIB was 1.148 (1.043–1.264) (p = 0.005). Similarly, OR values of 1.047 (1.020–1.074) for Group IIA and 1.118 (1.070–1.167) for Group IIB were determined for each degree Celsius increase in temperature and were statistically significant (p = 0.001 and p<0.001, respectively). The multivariable regression model revealed that the temperature increase was effective in predicting both Group IIA and Group IIB [p = 0.001 and p = 0.006, respectively (Table 4)].

**Table 4:** The effect of weather events on the number of patient visits.

	Univariable analysis	p-value	Multivariable analysis	p-value
	OR (95% CI)		OR (95% CI)	
Sunshine (hours)				
Group 1	reference			
Group 2A	1.062 (1.000–1.128)	0.050		
Group 2B	1.148 (1.043–1.264)	0.005		
Daily Average Actual Pressure (hPa)				
Group 1	reference			
Group 2A	0.983(0.928–1.041)	0.561		
Group 2B	0.986 (0.905–1.074)	0.754		
Daily Average Cloudiness (8 Octa)				
Group 1	reference			
Group 2A	0.937 (0.843–1.042)	0.229		
Group 2B	0.888 (0.755–1.044)	0.150		
Daily Average Relative Humidity (%)				
Group 1	reference			
Group 2A	0.987 (0.973–1.001)	0.071		
Group 2B	0.955 (0.934–0.977)	<0.001		
Daily Average Wind Speed (m/s)				
Group 1	reference			
Group 2A	1.328 (0.935–1.907)	0.124		
Group 2B	1.946 (1.208–3.136)	0.006		
Daily Average Temperature (°C)				
Group 1	reference			
Group 2A	1.047 (1.020–1.074)	0.001	1.093 (1.039–1.150)	0.001
Group 2B	1.118 (1.070–1.167)	<0.001	1.124 (1.033–1.222)	0.006
Daily Total Global Insolation Radiation (Kwatt/m^2^)>200000				
Group 1	reference			
Group 2A	1.763 (1.077–2.886)	0.024		
Group 2B	4.041 (1.737–9.401)	0.001		

## 4. Discussion

In this study, a positive correlation was observed between the increase in temperature, sunshine hours, and low humidity rates and the number of acute urticaria cases admitted to the emergency department. Accordingly, it was determined that the number of patients who applied to the emergency department with acute urticaria in the summer months when the temperature and sunshine duration was high and, on the days when there was low humidity, was high.

Zhang J. et al. investigated the relationship between 6-year outpatient visits of urticaria patients in Lanzhou, meteorological data, and air pollution and demonstrated that the daily average temperature increased the outpatient visits of urticaria patients.It was observed that the number of female patients was higher in urticaria admissions. High temperatures have been suggested to increase the risk of urticaria for people aged 0–14 and 15–59 years, while low temperatures increase the incidence of urticaria for people over 60 years of age [11]. Our study demonstrated no significant difference between the patient group aged 18–60 years and the patient group over 60 years old in terms of meteorological variables. However, the rate of urticaria (58%) was more common in females.

Losappio L et al. detected triggering factors in 42% of patients who presented to the emergency department with acute urticaria, whereas drugs were detected in 20.7%, insect bites in 10.2%, food in 7.4%, and contact urticaria in 3.7%; 58% of the cases did not have a triggering factor [12]. In the present study, this rate was 56.87%. When we investigated the effect of meteorological factors in cases in which a triggering factor could not be determined, sunshine hours, temperature, and humidity were indicated to have an impact. Another study by Nie Y. et al. revealed that high temperatures and low humidity increased the outpatient visits of urticaria patients. In addition, it was demonstrated that the risk of applying to the urticaria outpatient clinic was higher in men and in the patient group under 18 years of age [13]. Similar results were obtained in this study as well.

In a study performed in Brazil, where meteorological factors were examined in the etiology of acute asthma attacks in allergic asthma—which is similar to the pathophysiology of urticaria—temperature variability of 0–7 days was examined, and each 1°C increase was associated with a 1.0% increase in hospitalizations for asthma (95% CI; 0.7–1.4%) [14]. Another study from the People’s Republic of China revealed that 0- to 5-year-old children and men were more vulnerable to low temperatures and patients with asthma to barometric pressure. It has also been suggested that wind speed and humidity are protective factors for childhood asthma, and children aged 6–14 are more sensitive to atmospheric changes [15]. As a common result of the literature, temperature dropshave a negative impact on asthma risk, especially in children and low-latitude regions. The authors stated that this effect was because of an increase in lower respiratory tract infections [16].

A study determined that asthma exacerbations were more frequent when the air humidity increased in patients over 65 years of age. The authors considered that this effect might be because of the increase in air pollutant particles [17]. The present study demonstrated that low humidity rates caused an increase in admissions to the emergency department with acute urticaria.

In a meta-analysis of 16 studies, 31% (5/16) of studies indicated that temperature was the only factor associated with asthma hospitalization or emergency room admissions. In six studies (37%), both temperature and relative humidity were related to hospitalization [18]. In this study, it was determined that the number of patients who applied to the emergency department with acute urticaria, which has similar features to allergic asthma, was higher at increased temperature and low humidity.

## 5. Conclusion

In this study, in which we examined the effect of meteorological factors in patients who applied to the emergency department with acute urticaria and had no suspected etiological factors, we found that sunshine hours, temperature, and humidity rates were effective. There was an increase in the number of applications to the emergency service in the summer months when the sunshine hours were high and on the days when the high temperatures and low humidity rates. When the clinician cannot detect the triggering factor in patients who present to the emergency department with acute urticaria, we recommend that the meteorological factors be evaluated.

## 6. Limitations

This study was based on data applied to a third-level hospital in our city and was a single-center study. Only patients who applied to our hospital from the city center were included in our study. Patients admitted from rural areas could not be enrolled.

## Supporting information

S1 Data (XLSX)
